# A Two-Gene Signature, *SKI* and *SLAMF1*, Predicts Time-to-Treatment in Previously Untreated Patients with Chronic Lymphocytic Leukemia

**DOI:** 10.1371/journal.pone.0028277

**Published:** 2011-12-14

**Authors:** Carmen D. Schweighofer, Kevin R. Coombes, Lynn L. Barron, Lixia Diao, Rachel J. Newman, Alessandra Ferrajoli, Susan O'Brien, William G. Wierda, Rajyalakshmi Luthra, L. Jeffrey Medeiros, Michael J. Keating, Lynne V. Abruzzo

**Affiliations:** 1 Department of Hematopathology, The University of Texas M.D. Anderson Cancer Center, Houston, Texas, United States of America; 2 Department of Biostatistics and Applied Mathematics, The University of Texas M.D. Anderson Cancer Center, Houston, Texas, United States of America; 3 Department of Leukemia, The University of Texas M.D. Anderson Cancer Center, Houston, Texas, United States of America; University of Barcelona, Spain

## Abstract

We developed and validated a two-gene signature that predicts prognosis in previously-untreated chronic lymphocytic leukemia (CLL) patients. Using a 65 sample training set, from a cohort of 131 patients, we identified the best clinical models to predict time-to-treatment (TTT) and overall survival (OS). To identify individual genes or combinations in the training set with expression related to prognosis, we cross-validated univariate and multivariate models to predict TTT. We identified four gene sets (5, 6, 12, or 13 genes) to construct multivariate prognostic models. By optimizing each gene set on the training set, we constructed 11 models to predict the time from diagnosis to treatment. Each model also predicted OS and added value to the best clinical models. To determine which contributed the most value when added to clinical variables, we applied the Akaike Information Criterion. Two genes were consistently retained in the models with clinical variables: *SKI* (v-SKI avian sarcoma viral oncogene homolog) and *SLAMF1* (signaling lymphocytic activation molecule family member 1; CD150). We optimized a two-gene model and validated it on an independent test set of 66 samples. This two-gene model predicted prognosis better on the test set than any of the known predictors, including ZAP70 and serum β2-microglobulin.

## Introduction

Gene expression profiling studies of CLL using microarray technology have examined differential gene expression with respect to a wide variety of prognostic factors, including the somatic mutation status of the immunoglobulin heavy chain variable region (*IGHV*) genes, clinical stage, cytogenetic abnormalities, and others [1], [2], [3], [4], [5], [6], [7]. These studies have shown that CLL cases share a characteristic gene expression profile, and that prognostic subtypes are distinguishable by small subsets of genes. Gene expression profiling results also have yielded lists of genes that are potential biomarkers of prognosis, many of which have shown promise in the clinical setting. For example, expression of ZAP70 has been found to correlate with unmutated *IGHV* somatic mutation status and a poor clinical outcome [3], [4], [8], [9], [10], [11]. These studies have focused on different prognostic factors. Thus, it is not surprising that they have identified independent sets of potential biomarkers, which need to be validated in order to be clinically useful. Towards this goal, in a previous study we developed and validated predictive models of prognosis in untreated CLL patients. We first produced a quantitative real-time polymerase chain reaction assay on microfluidics cards (MF-QRT-PCR) using a panel of candidate biomarkers linked to *IGHV* somatic mutation status. These markers were selected by re-analyzing raw data from previously published microarray studies [1], [2], [3], [4], [12]. We then applied this assay to untreated CLL patient samples, and demonstrated that we could predict *IGHV* somatic mutation status with 90% accuracy based on the expression of as few as three genes [13].

In the current study, we sought to develop and validate predictive models based on a wide range of reported clinical, cytogenetic, and molecular prognostic markers, including *IGHV* somatic mutation status. We began by re-analyzing previously published microarray studies [5], [6], [7], [14], [15], [16], [17], [18]. Our study group was 131 previously-untreated CLL patients for whom samples were available for analysis. We first identified the best clinical models to predict time-to-event outcomes, i.e., time-to-treatment (TTT) and overall survival (OS), in a training set of 65 samples. We then sought to identify individual genes or combinations of genes, in the training set, in which expression was related to prognosis. By performing extensive cross-validation of both univariate and multivariate models to predict TTT, we identified four sets of genes, ranging from 5 to 13 genes that could be used to construct multivariate prognostic models. By applying statistical methods to optimize each of these four sets of genes on the training set, we constructed 11 models to predict the interval between diagnosis and first treatment. Each model also predicted OS and added value to the best clinical models. To determine which genes contributed the most value when added to the clinical variables, we applied the Akaike Information Criterion [19]. Two genes were consistently retained in the models in the presence of the clinical variables: *SKI* (v-SKI avian sarcoma viral oncogene homolog) and *SLAMF1* (signaling lymphocytic activation molecule family member 1; CD150). We then optimized a two-gene predictive model and validated its performance in an independent test set of 66 CLL patient samples. This model was validated successfully, and predicted prognosis on the test set better than any of the existing clinical predictors.

## Results

### Patient characteristics

The characteristics of 131 previously untreated CLL patients are summarized in **Table 1**. We collected the following clinical and laboratory parameters: age at diagnosis, gender, Rai stage, bulky lymphadenopathy (splenomegaly ≥8 cm below the costal margin or lymph nodes ≥5 cm), white blood cell (WBC), prolymphocyte, and platelet counts, hemoglobin level, serum lactate dehydrogenase (LDH) and β2-microglobulin levels, hypogammaglobulinemia, CLL score [20], surface immunoglobulin (IG) light chain isotype, CD38, *IGHV* somatic mutation status, ZAP70 protein expression, and cytogenetic complexity (defined as ≥3 abnormalities). With the exception of the WBC count, there were no statistically significant differences between the 65 samples used for the training set and the 66 samples used for the validation set. The median time from diagnosis to sample collection for the 131 patients was 28 months (range 1–211 months); there was no significant difference (p = 0.77) in time from diagnosis to sample collection between the training set (median 26 months) and the test set (median 29 months, **Table 1**).

**Table 1:** Clinical and Laboratory Features.*

		All Patients(n = 131)	Training(n = 65)	Test Set(n = 66)	p value†
**Age at diagnosis (years)**	**Median, years** **(range)**	56.7(26.7, 80.9)	56.1(26.7, 80.1)	58.5(37.6, 80.9)	0.12
**Gender**	**Male, n (%)** **Female, n (%)**	81 (61.8)50 (38.2)	42 (64.6)23 (35.4)	39 (59.1)27 (40.9)	0.59
**Rai stage**	**0–2, n (%)** **3–4, n (%)**	102 (77.9)29 (22.1)	54 (83.1)11 (16.9)	48 (72.7)18 (27.3)	0.21
**Splenomegaly** **(cm below costal margin)**	**<8 cm, n (%)** **≥8 cm, n (%)**	126 (95.5)5 (4.5)	63 (96.9)2 (3.1)	63 (95.5)3 (4.5)	0.99
**WBC count** ‡	**≤150×10^9^/L, n (%)** **>150×10^9^/L, n (%)**	118 (90.1)13 (9.9)	63 (96.9)2 (3.1)	55 (83.3)11 (16.7)	0.02
**Hgb (g/dL)** ‡	**Median** **(range)**	12.9(8.2, 17.4)	12.9(10.0, 17.0)	12.9(8.2, 17.4)	0.59
**Prolymphocytes (% of WBC)**	**Median** **(range)**	3.0(0.0, 21.0)	4.0(0.0, 21.0)	2.5(0.0, 15.0)	0.19
**Platelets (×10^9^/L)** ‡	**Median** **(range)**	168(39, 476)	168(39, 379)	169(60, 476)	0.31
**LDH (U/L)** ‡	**Median** **(range)**	526(274, 1818)	527(338, 1313)	517(274,1818)	0.10
**β2M (mg/L)** ‡ (n = 130)	**<4, n (%)** **≥4, n (%)**	98 (75.4)32 (24.6)	45 (70.3)19 (29.7)	53 (80.3)13 (91.7)	0.22
**Serum Ig** ‡ (n = 118)	**Decreased, n (%)** **Normal, n (%)**	69 (58.5)49 (41.5)	30 (52.6)27 (47.4)	39 (63.9)22 (36.1)	0.26
**CLL score** (n = 113)	**Atypical, n (%)** **Typical, n (%)**	32 (28.3)81 (71.7)	18 (31.6)39 (68.4)	14 (25.0)42 (75.0)	0.53
**Surface IgL** (n = 125)	**Kappa, n (%)** **Lambda, n (%)**	78 (62.4)47 (37.6)	36 (60.0)24 (40.0)	42 (64.6)23 (35.4)	0.71
**CD38** ‡ (n = 124)	**Low, n (%)** **High, n (%)**	98 (79.0)26 (21.0)	50 (83.3)10 (16.7)	48 (75.0)16 (25.0)	0.28
***IGHV*** ** SM status** (n = 130)	**Mutated, n (%)** **Unmutated, n (%)**	67 (51.5)63 (48.5)	36 (55.4)29 (44.6)	31 (47.7)34 (52.3)	0.48
**ZAP70** (n = 113)	**Positive, n (%)** **Negative, n (%)**	62 (54.9)51 (45.1)	32 (59.3)22 (40.7)	30 (50.8)29 (49.2)	0.45
**Karyotype** ‡ (n = 94)	**Simple, n (%)** **Complex, n (%)**	82 (87.2)12 (12.8)	41 (93.2)3 (6.8)	41 (82.0)9 (18.0)	0.13
**Time of follow up from diagnosis**	**Median, months** **(range)**	101.3(9.6, 271.3)	109.2(9.6–252.4)	93.1(28.3–271.3)	0.46
**Time from diagnosis to sample**	**Median, months** **(range)**	27.5(0.7–211.5)	26.0(0.7–151.9)	29.2(1.0–211.5)	0.77
**Time from diagnosis to first treatment**	**Median, months** **(range)**	28.8(0.7, 211.5)	28.0(0.7–198.3)	30.7(1.0–211.5)	0.84
**Overall survival at final follow up**	**n (%)**	100 (76.3)	45 (69.2)	55 (83.3)	0.10

**Abbreviations**: WBC, white blood cell; Hgb, hemoglobin; Prolymphs, prolymphocytes; LDH, serum lactate dehydrogenase; β2M, serum β2 microglobulin; serum Ig, serum immunoglobulin levels; surface IgL, surface immunoglobulin light chain isotype; *IGHV* SM status, immunoglobulin heavy chain variable region gene somatic mutation status.

*Rai stage, splenomegaly, WBC count, Hgb, Prolymphs, Platelets, LDH, β2M, serum Ig were determined at the time the sample was obtained. The CLL score, surface IgL, *IGHV* SM status, ZAP70, and karyotype were determined on samples obtained before treatment was initiated.

† All p values were calculated using the two-sided Fisher's Exact test except for age in years, which was calculated using the two-sided t-test, and time-to-event parameters (log-rank test).

‡ The normal ranges are: WBC, 4–11×10^9^/L; Hgb, 14.0–18.0 g/dL, platelets, 140–440×10^9^/L; LDH, 313–618 U/L; β2M, 0.7–1.8 mg/L; serum Ig, IgM 29–214 g/dL, IgA 74–327 g/dL, IgG 624–1680 g/dL. Serum Ig are considered decreased (hypogammaglobulinemia) if ≥2 immunoglobulin fractions are below the normal range. CD38 is low if <30% of CD19+ cells express CD38, and high if ≥30% of CD19+ express CD38. Karyotypes are considered simple if the number of abnormalities is <3, and complex if the number is ≥3.

### Clinical Predictors of TTT and OS in CLL

A flow diagram of the complete statistical analysis is presented in **Figure S1**. We first sought to identify the best clinical models to predict time-to-event outcomes in the training set of 65 samples assayed on cards A and B. In order to determine the best model that we could construct from pre-existing markers, we analyzed all of the clinical and laboratory variables listed in **Table 1**. We performed time-to-event analyses using two different starting points: time of diagnosis and time of sample collection. We also used two different endpoints: TTT and OS. Based on these starting and endpoints, we constructed four models. We found that the best model to predict the time from diagnosis to treatment incorporated the (log) serum LDH level, IG light chain isotype, and platelet count. We refer to the numerical predictions from this model as the “clinical score”. The best model to predict time from sample collection to treatment incorporated the *IGHV* somatic mutation status, IG light chain isotype, platelet count, WBC count, and (log) serum β2-microglobulin level. The best model to predict OS from diagnosis incorporated age at diagnosis and *IGHV* somatic mutation status. The best model to predict OS from sample collection incorporated age at diagnosis, *IGHV* somatic mutation status, and (log) serum β2 microglobulin level. After we constructed these clinical models, we assessed how MF-QRT-PCR assay data might add to the accuracy of the predictions. (The complete computer scripts used to analyze the data are available at http://bioinformatics.mdanderson.org/Supplements/Microfluidics/Prognosis.) [21].

### Identification of Gene Markers of Prognosis (Feature Selection)

Our next goal was to identify individual genes or combinations of genes that were related to prognosis in the training set. We began by re-analyzing the data from cards A and B in light of the updated clinical data, but focused on genes that had been chosen for inclusion on the validation card C. In order to identify robust prognostic markers of TTT, we performed four univariate analyses (one gene at a time) on the training data from the 65 patients assayed on cards A and B. We performed analyses using two starting points: diagnosis and sample collection. We incorporated gene expression either as a continuous predictor of outcome or as a dichotomous variable. In univariate models, we found at least 15 genes that predicted TTT (**Table 2**). For the most part, the same genes were significant predictors under all four conditions: time from diagnosis, gene expression as a dichotomous variable; time from diagnosis, gene expression as a continuous variable; time from sample collection, gene expression as a dichotomous variable; time from sample collection, gene expression as a continuous variable.

**Table 2:** Ability of genes to predict time-to-treatment on the training set.

	Cox proportional hazards				Cross-validation		
	Log-rank p-values				% of times selected		
	Time from Diagnosis		Time from Sample Collection			Diagnosis	Sample
	Dichotomous	Continuous	Dichotomous	Continuous	Univariate	Multivariate	
SKI	**0.00070**	**0.00153**	**0.00054**	**0.00040**	96.7	68.3	77.3
NT5C2	**0.00327**	**0.00932**	**0.00045**	**0.00142**	91.3	34.0	43.3
AICDA	**0.00329**	**0.02869**	**0.00059**	**0.00595**	83.7	13.3	14.3
SLAMF1	**0.03603**	**0.00390**	**0.02166**	**0.00032**	82.7	45.3	26.0
CD14	**0.01806**	**0.01375**	**0.00229**	**0.00033**	76.3	12.7	58.3
FGL2	0.05126	**0.02619**	**0.00104**	**0.00050**	75.7	18.3	23.7
NUDC	**0.01311**	**0.01185**	**0.01277**	**0.00844**	58.7	16.0	8.0
NRIP1	0.09115	**0.01747**	**0.00887**	**0.00047**	53.3	7.0	26.0
EGR3	0.11961	**0.01697**	**0.02460**	**0.00120**	47.0	15.0	9.7
OAS3	**0.03989**	**0.01868**	**0.02203**	**0.00527**	43.7	18.0	40.7
MLXIP	**0.01028**	**0.00922**	**0.03763**	**0.02238**	41.3	23.7	7.7
TPST2	**0.02604**	0.11673	**0.00166**	**0.01538**	39.3	8.7	6.7
GZMK	0.10461	0.47112	**0.00425**	**0.01093**	19.7	3.7	19.0
TRIB2	**0.04825**	**0.03755**	**0.03220**	**0.03645**	18.7	4.3	3.3
BLNK	0.21545	**0.01056**	0.17736	**0.01136**	15.3	10.7	9.7
ATF4	**0.03024**	**0.04587**	0.06964	0.14722	9.0	8.3	3.7
ZAP70	0.05281	0.72270	**0.01290**	0.20473	3.0	1.3	0.3
CCL5	0.31346	0.20183	0.11853	0.10792	2.7	2.7	2.7
FLNB	0.33042	0.16040	0.12236	0.07366	2.0	1.0	0.0
FGFR1	0.20586	0.13564	0.19548	0.22199	2.0	2.0	2.0
ZBTB20	0.32892	0.26458	0.08767	0.05625	1.0	0.7	1.0
GFI1	0.12424	0.27589	0.11658	0.23475	0.7	0.0	0.0
ATRX	0.53365	0.21520	0.23976	0.12142	0.3	0.3	0.3
SEPT10	0.84619	0.57552	0.61241	0.07739	0.3	0.0	0.3
LPL	0.30342	0.19845	0.19664	0.08380	0.3	0.0	0.0
WSB2	0.42500	0.97415	0.15628	0.38180	0.0	0.0	0.0
TNFRSF8	0.60838	0.69192	0.28498	0.52499	0.0	0.0	0.0
RIOK2	0.83187	0.30138	0.96935	0.16604	0.0	0.0	0.0
P2RX1	0.28422	0.20501	0.15201	0.06186	0.0	0.0	0.0
LDOC1	0.48194	0.97673	0.15223	0.62795	0.0	0.0	0.0
LASS6	0.72274	0.72844	0.48064	0.52066	0.0	0.0	0.0
CRY1	0.27369	0.44715	0.12731	0.23697	0.0	0.0	0.0
COBLL1	0.59439	0.53532	0.77744	0.86219	0.0	0.0	0.0
CD86	0.94053	0.82897	0.84059	0.18854	0.0	0.0	0.0
BCL7A	0.99125	0.79130	0.46758	0.57526	0.0	0.0	0.0
BANK1	0.98999	0.36153	0.60229	0.81261	0.0	0.0	0.0
ANXA2	0.55803	0.44614	0.79069	0.65462	0.0	0.0	0.0

We also used the training data to cross-validate univariate and multivariate models to predict TTT. We repeatedly and randomly selected 50 of the 65 training samples and used them to fit univariate models. Genes for which the geometric mean of the four univariate p-values was <0.03 were selected for inclusion in a multivariate model that combined continuous gene expression levels to predict the time from diagnosis to treatment or time from sample collection to treatment. We used AIC to decide which genes to retain in the best multivariate models (**Table 2**). We identified two genes retained in ≥25% of the cross-validation multivariate models to predict time from diagnosis to treatment, *SKI* and *SLAMF1*. Six genes, *SKI*, *CD14*, *NT5C2*, *OAS3*, *NRIP1*, and *SLAMF1*, were retained in ≥25% of the cross-validation multivariate models to predict time from sample collection to treatment.

We repeated this analysis using OS as the outcome instead of TTT (**Table S1**). The ability of individual genes to predict OS was weak, with only four genes (*CD14*, *WSB2*, *TNFRSF8*, and *NT5C2*) being significant in ≥10% of the cross-validation univariate models. In general, significance levels for the ability to predict OS were lower than those for TTT because there were many fewer events. After a median follow up of more than 8 years (101 months, range 10–271 months) from diagnosis, out of 131 patients 109 had required treatment and 31 had died (**Table 1**). It is possible with additional follow-up that we would be able to predict OS. In this analysis, we did not take into account clinical covariates that are associated with prognosis in previously untreated CLL patients. Thus, we repeated the analysis in order to determine which genes added predictive power to the best clinical models, described above. After accounting for known clinical factors, we found no single gene that added to our ability to predict either TTT or OS. However, the genes *SLAMF1*, *NRIP1*, *SKI*, *NT5C2*, *FGFR1*, *CD14*, and *CRY1* showed borderline statistical significance in the training set, depending on the start time (diagnosis or sample collection) or endpoint (treatment or survival). This finding suggested that appropriate combinations of gene expression might provide prognostic value that added to the existing clinical variables.

### Building Gene-Based Models of Prognosis

**Figure 1:**
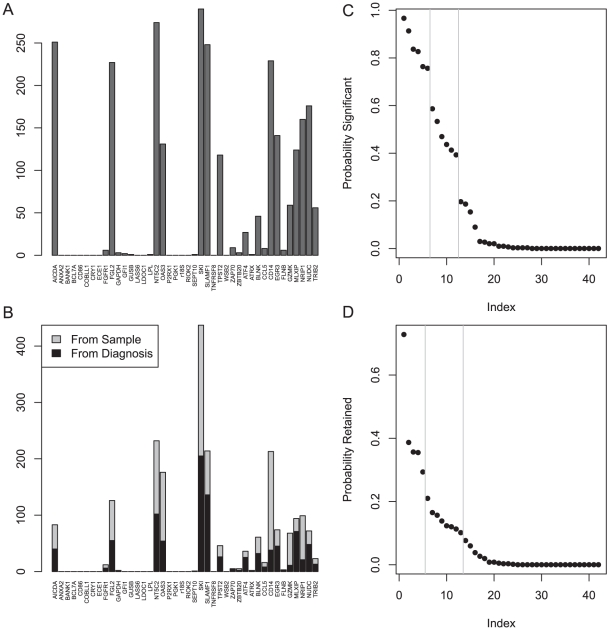
Cross-validation of gene predictors on the training set. (A) Histogram of the number of times each gene was statistically significant among 300 assessments of univariate models. (B) Histogram of the number of times each gene was retained in a multivariate model to predict time-to-treatment either from diagnosis or from sample collection (300 each). (C) Sorted probabilities that a gene was significant in a univariate model. Gaps in the figure identify a six-gene and a twelve-gene subset. (D) Sorted probabilities that a gene was retained in a multivariate model. Gaps in the figure identify a five-gene and a thirteen-gene subset.

By evaluating the number of times a gene was selected as significant in the cross-validation analyses performed above, we identified four sets of genes, containing 5, 6, 12, or 13 genes, that could be used to construct multivariate prognostic models (**Figure 1**). Starting with each of these sets of genes, we constructed Cox proportional hazards models to predict the time from diagnosis to treatment. We used both AIC and BIC to build optimal models that used the fewest number of genes from each set. We repeated the process starting from the time of sample collection. This process produced 11 different models (**Table 3**), each of which was highly significant for predicting the time from diagnosis to treatment on the training set.

**Table 3:** Genes retained in multivariate models to predict time from diagnosis to treatment in the training dataset.

Gene Set	M6	M6	M6,SAM‡	M5	M6,SAM‡	M5*	M12	M6*	M13	M12*	M13*
Optimizer†	AIC	BIC	AIC	AIC,BIC	BIC		AIC,BIC		AIC,BIC		
SKI	Yes (c)	Yes (c)	Yes	Yes (c)	Yes (c)	Yes (c)	Yes (c)	Yes (c)	Yes (c)	Yes (c)	Yes
NT5C2	Yes	Yes	Yes	Yes	Yes	Yes	–	Yes	–	Yes	Yes
SLAMF1	–	Yes (c)	–	–	Yes (c)	Yes (c)	Yes (c)	Yes (c)	Yes (c)	Yes (c)	Yes (c)
CD14	–	–	Yes	–	Yes	Yes	–	Yes	–	Yes	Yes
FGL2	–	–	–	–	–	–	Yes	Yes	Yes	Yes	Yes
OAS3	–	–	–	Yes	–	Yes	–	–	–	Yes	Yes
NUDC	–	–	–	–	–	–	Yes	–	Yes	Yes	Yes
MLXIP	–	–	–	–	–	–	Yes	–	Yes	Yes	Yes
AICDA	–	–	–	–	–	–	–	Yes	–	Yes	Yes
NRIP1	–	–	–	–	–	–	–	–	–	Yes	Yes
EGR3	–	–	–	–	–	–	–	–	–	Yes	Yes
BLNK	–	–	–	–	–	–	–	–	Yes	–	Yes
TPST2	–	–	–	–	–	–	–	–	–	Yes	–
GZMK	–	–	–	–	–	–	–	–	–	–	Yes (c)
**Predicts OS?** §	**0.0234**	**0.0233**	**0.0036**	**0.0139**	**0.0094**	**0.0048**	**0.0383**	**0.0132**	**0.0473**	**0.0087**	**0.0307**
**Adds to CM?** ¶	**0.034**	**0.016**	**0.031**	**0.030**	**0.015**	**0.016**	**0.011**	**0.012**	**0.0046**	**0.0031**	**0.0027**

**Abbreviations**: M, model; AIC, Akaike Information Criterion; BIC, Bayes Information Criterion; SAM, sample collection; CM, clinical model.

*Four different sets of genes were chosen as starting points, containing 5, 6, 12, or 13 genes.

† We applied stepwise forward-backward methods to each gene set to optimize either AIC or BIC.

‡ Two gene sets optimized predictions of the time from sample collection to treatment; all others looked at the time from diagnosis to treatment.

§ Log rank P value to test, via Cox proportional hazards, if a continuous score derived to predict time-to-treatment also predicts overall survival.

¶ P-value computed from a chi-squared test of whether the continuous score adds value to the existing clinical predictors.

(c) Individual genes that remained significant when added to the existing clinical predictors.

We used each model to compute a gene prognostic (GP) score for each patient, as a linear combination of gene expression values (**Table S2**). In general, the GP scores were strongly correlated from one model to another. The GP scores were also highly significant for predicting the time from sample collection to treatment on the training set. We evaluated the GP scores from each model for their ability to predict OS. As continuous variables, the GP scores from all 11 models were significant for predicting OS (**Table 3**).

Gene prognostic scores were independent of the best clinical model of prognosis described above and, therefore, contributed additional information (**Table 3**). To determine which genes in each GP score contributed the most value when added to the clinical variables, we again applied AIC to models that added the gene sets to the clinical variables in order to predict time from diagnosis to treatment or OS. The only genes that were consistently retained in the models, in the presence of the clinical variables, were *SKI* and *SLAMF1*. Both genes were expressed in all samples, but at differing levels with respect to time from diagnosis to treatment.

### Training a Two-Gene Prognostic Model

Since *SKI* and *SLAMF1* were the genes that were most consistently retained as predictors of time from diagnosis to treatment in the presence of clinical variables, we developed and tested a two-gene model based on *SKI* and *SLAMF1* for predicting TTT or OS. We computed the GP score for the two-gene model from the ΔΔ-Ct values for the genes *SKI* and *SLAMF1* using the formula: GP score = 0.847 *SKI*+0.158 *SLAMF1*. To dichotomize the score, we defined a cutoff at the median value (0.022) on the training samples. Scores greater than the median were considered high; scores less than or equal to the median were considered low. When applied to the training set, we found that the GP score based on the two-gene model using *SKI* and *SLAMF1* effectively predicted both time from diagnosis to treatment (p = 0.000127), and time from sample collection to treatment (p = 9.08×10^−6^). The GP score also showed the correct trend to predict OS from the time of diagnosis, (p = 0.0554).

### Validation of a Two-Gene Prognostic Model

**Figure 2:**
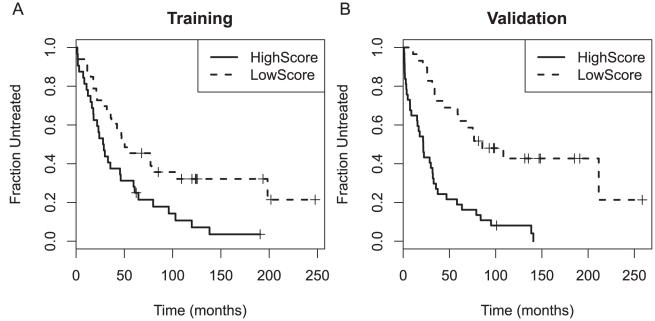
Kaplan-Meier plots of the time from diagnosis to treatment stratified by the gene prognostic (GP) score (a linear combination of the expression of *SKI* and *SLAMF1*) in (A) the training set and (B) the validation set.

Based on these results, we sought to determine if the GP score based on the two-gene model effectively predicted time from diagnosis to treatment in an independent test set of 66 samples. We found that the two-gene model effectively predicted time from diagnosis to treatment, both as a continuous score (p = 1.78×10^−8^) and as a dichotomous variable (p = 6.47×10^−6^) (**Figure 2**). The same GP score predicted OS after diagnosis on the validation dataset as a dichotomous variable (p = 0.0182), but was only marginally significant as a continuous variable (p = 0.0994). Moreover, the same GP score also predicted time from sample collection to treatment, both as a continuous variable (p = 0.00098) and as a dichotomous variable (p = 0.00164).

**Figure 3:**
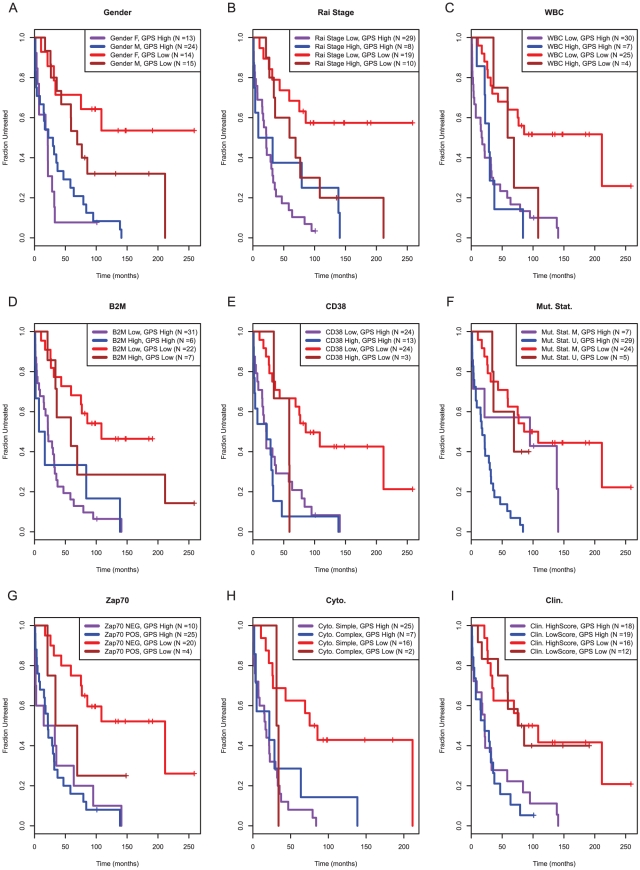
Kaplan-Meier plots of time from diagnosis to treatment in the validation set, showing the interactions between the *SKI*-*SLAMF1* score and (A) gender, (B) Rai stage, (C) WBC count, (D) serum β2-microglobulin level, (E) CD38, (F) *IGHV* somatic mutation status, (G) ZAP70, (H) cytogenetic complexity, and (I) clinical score.

Finally, we sought to determine if the two-gene model added to the clinical model in an independent test set. Because we had found previously that the clinical score was a poor predictor of TTT in this data set, we re-fit the clinical model on the test set and then compared it to the GP score. The GP score, derived from the training data, remained the best predictor of TTT, although the platelet count provided additional prognostic ability. We also compared the GP score to individual clinical predictors. The GP score was at least as effective as *IGHV* somatic mutation status at predicting TTT, and the GP score more effectively predicted TTT than any of the other variables in **Table 1**, including Rai stage, gender, serum β2 microglobulin level, WBC count, CD38 or ZAP70 expression, and cytogenetic complexity (**Figure 3**).

## Discussion

The goal of this study was to develop and validate models of gene expression that would contribute prognostic information to predict TTT for patients with previously untreated CLL. This study has yielded a two-gene model that adds prognostic information independent of and in addition to known clinical data.

We analyzed all data using an extremely rigorous training and validation design. Extensive model building, with cross-validation, was applied to the training data set of 65 samples. To avoid over-fitting, we tested only the best gene-expression-based model (the model with *SKI* and *SLAMF1*) on an independent set of 66 samples that had been assayed on a new printing (Card C) of the microfluidics cards. The highly significant p-values on this independent set provide powerful evidence supporting the clinical utility of the two-gene model. We applied similar rigorous statistical methods to the clinical data. For these analyses, we used standard statistical methods, i.e., Cox proportional hazards with stepwise AIC to choose the variables that should be retained in the model. Somewhat surprisingly, the predictive model based purely on the clinical variables did not validate; this result, unfortunately, is one of the possible consequences whenever one assesses combinations of many variables to construct predictive models, and is the reason that separate training and validation datasets are required. However, because numerous clinical factors have been shown previously to be significant predictors of prognosis in many studies of patients with CLL, we extended our analysis to pose a greater challenge to the two-gene model.

Using the two-gene model that we learned from the training set to make predictions (GP scores) on the validation set, we compared the predictions from the two-gene model to each of the individual clinical factors listed in **Table 1**. Even in this setting, the two gene-model predicted TTT in the validation set better than all other factors, except, possibly, for *IGHV* somatic mutation status. While further studies will be necessary to determine whether mutation status or the two-gene model is ultimately a better predictor of TTT in CLL patients, our results on the independent validation set establish *SKI* and *SLAMF1* expression as powerful predictors of prognosis in previously untreated patients with CLL.

In order to identify candidate biomarkers of prognosis, we chose genes that had been identified either in previous gene expression profiling studies that we performed or from our review of the literature. However, *SKI* was identified serendipitously. The goals of our initial studies were to evaluate the MF-QRT-PCR assay technology, and to develop statistical methods to normalize and analyze the data [1], [13], [14]. Studies indicate that no single housekeeping gene works well in all studies, and that better results are obtained by using multiple housekeeping genes for normalization [14], [22]. Based on an initial experiment using samples obtained from nine previously-untreated CLL patients (four with unmutated and five with mutated *IGHV*), we selected eight potential control genes that appeared to show little variability across the samples, one of which was *SKI* [14]. However, in a subsequent study of 29 CLL samples (14 mutated and 15 unmutated), *SKI* was found to vary significantly across the samples [13]. Thus, it was eliminated from our list of endogenous control genes, but was retained on Card A and included in our analysis as a candidate biomarker of prognosis.

Our studies indicate that higher expression levels of *SKI* and *SLAMF1* mRNA are associated with a longer TTT in patients with previously untreated CLL. *SKI*, located on chromosome 1p36.3, encodes the nuclear protein homolog of the avian sarcoma viral (v-ski) oncogene [23], [24]. *SKI* is expressed ubiquitously at low levels in adult and embryonic tissues [24], [25]. During embryogenesis *SKI* appears to regulate cellular differentiation, particularly of neural tissues and skeletal muscle. In adult tissues, *SKI* participates in diverse cellular functions including proliferation, differentiation, cell cycle progression, and apoptosis. *Ski* also has been reported to be relatively overexpressed in murine memory B cells compared germinal center B cells, suggesting that it contributes to memory B cell differentiation [26]. Depending upon the context, overexpression of *SKI* can result in either transformation or terminal differentiation [25]. Thus, *SKI* can function either as a proto-oncogene or as a tumor suppressor gene. These divergent functions likely reflect its ability to interact with a variety of transcription factor partners [25]. Consistent with its role as an oncogene, SKI expression is increased in different cancer types, including esophageal squamous cell carcinoma [27], melanoma [28], and acute myeloid leukemias [29], [30]. One of its most important mechanisms of action is to negatively regulate TGF-β signaling by interacting with Smad proteins and repressing their transcriptional activity [31]. *SKI* also blocks differentiation by inhibiting RARα signaling in some subtypes of acute myeloid leukemia [29], [30]. However, *SKI* may also function as a tumor suppressor gene [23]. Shinagawa and colleagues demonstrated that *Ski*-deficient heterozygous mice developed hematologic malignancies, following challenge with a chemical carcinogen, predominantly T-cell lymphomas, but also B-cell lymphoma and myeloid leukemias [23]. They hypothesized that the tumor suppressor activity of SKI results from its ability to mediate transcriptional repression by other known tumor suppressors, namely Mad and Rb, which interact with multiple target genes to negatively regulate cell cycle progression. In this study, we found that increased *SKI* expression is associated with a better outcome, raising the possibility that it may function as a tumor suppressor gene in some cases of CLL.

*SLAMF1* (CD150) is one of a family of nine (*SLAMF1–9*) glycoprotein receptors that belong to the *IG* supergene family, all of which reside on chromosome 1q23 [32]. Members of this family regulate hematopoietic stem cell differentiation, leukocyte adhesion and activation, and humoral immune responses. In general, members of this family act as self-ligands. Most, including SLAMF1, are transmembrane proteins that contain tyrosine residues within their cytoplasmic domains. These tyrosine residues interact with adaptor proteins that connect the receptors to signal transduction networks. One of the adaptors, SLAM-associated protein (SAP), encoded by the *SH2D1A* gene, is mutated in patients with X-linked lymphoproliferative syndrome [33], [34], [35]. *SLAMF1* is expressed on thymocytes, memory T cells, B cell subsets, mature dendritic cells and platelets [32], [36], [37]. Its expression is rapidly induced on naïve T cells and B cells after activation [32], [36], [37]. It also serves as a cellular receptor for the measles virus [32]. *SLAM* family members are required for germinal center formation and for generation of humoral immune responses and memory B cells [32], [38], [39]. Studies of *SLAMF1* expression in human B-cell subsets have demonstrated that its expression varies with stage of differentiation [38], [40]. *SLAMF1* expression increases during B-cell development [40]. However, *SLAMF1* expression by memory B cells may vary depending upon the compartment studied. In human spleen, Good and colleagues [38] found that SLAMF1 was absent from memory B cells. In contrast, De Salort and colleagues [40] reported that SLAMF1 was highly expressed on memory B cells isolated from tonsil, but showed bimodal expression on splenic memory B cells.

In a previous meta-analysis of three gene expression profiling studies, we found that *SLAMF1* was relatively overexpressed in normal peripheral blood B cells compared to CLL cells [2], [3], [12]. We subsequently determined that *SLAMF1* is relatively overexpressed in mutated compared to unmutated untreated CLL patients [13]. Similarly, Mittal and colleagues [41] identified *SLAMF1* as one of 27 genes that was overexpressed in CLL cases with cytogenetic markers of good prognosis (del(13q) and normal) relative to cases with cytogenetic markers of poor prognosis (del(11q) and +12), as assessed by fluorescence in situ hybridization (FISH). Taken together, these findings suggest that signaling through *SLAMF1* may be dysregulated in CLL cells compared to normal peripheral blood B cells, and that the degree of dysregulation may be reflected in more aggressive clinical behavior.

As a first step in this study, we constructed clinical models of prognosis for our CLL study group. As expected, we found that the best models incorporated combinations of well-known markers of prognosis: age at diagnosis, WBC count, serum LDH and serum β2 microglobulin levels, and *IGHV* somatic mutation status. However, we were surprised to find that the best model to predict TTT from the time of sample collection also incorporated the IG light chain isotype. In particular, we found that patients with somatically-mutated kappa-positive CLL had a statistically significantly longer TTT (p = 0.00032) than the other three subgroups, i.e., unmutated kappa-positive, mutated lambda-positive, and unmutated lambda-positive CLL, which showed similar TTTs (data not shown). Stated differently, patients with somatically mutated lambda-positive CLL had a TTT that was similar to CLL patients with unmutated *IGHV*. An early study, which assessed surface IG expression by flow cytometry in untreated patients with B-cell non-Hodgkin lymphomas found that patients with lambda-positive CLL/small lymphocytic lymphoma had shorter survival than patients with kappa-positive CLL [42]. However, a much larger study performed several years later found no difference in survival between newly-diagnosed CLL patients whose cells expressed kappa compared to lambda [43]. Neither study evaluated TTT and both were based on older lymphoma classification schemes that lacked precision. Our patient cohort contained an equal number of IG kappa and lambda light chain expressing cases (62 cases each). The mutated cases were almost evenly divided between kappa (33 cases) and lambda (29 cases); the unmutated cases more often showed kappa expression (44 kappa-positive vs. 18 lambda-positive, p = 0.064). A similar kappa predominance among unmutated cases has been described previously [44]. Unlike that study, we did not find a lambda predominance in the mutated cases. One possible explanation that could account for the shorter TTT that we observed for somatically mutated lambda-positive CLL patients is that our patient cohort might contain an excess of cases that use *IGHV* families associated with a poor prognosis regardless of mutation status, i.e., *VH3-21*, *VH3-48*, and *VH3-*53 [45], [46]. Cases that use these families also tend to show a lambda light chain bias. However, of the 10 cases that used one of these *IGHV* families in our patient cohort, only four were somatically mutated and expressed lambda light chain. Thus, we are unable to account for the association of lambda immunoglobulin light chain expression and shorter TTT based on *IGHV* family use.

In summary, the goal of this study was to develop a clinically useful assay based on the expression of a limited set of protein coding genes to determine prognosis in patients with previously untreated CLL. The two-gene signature that we have identified is similar to other multi-gene panels (Onco*type* Dx® assays) that are currently used to predict the likelihood of chemotherapy benefit and recurrence risk in early stage breast cancer and the recurrence risk in colon cancer (http://www.genomichealth.com/OncotypeDX/Index.aspx). We believe this two-gene signature predicts TTT at least as well as current markers of prognosis in untreated CLL patients. Additional studies using this two-gene model performed on larger patient cohorts will be helpful to further assess clinical utility.

## Materials and Methods

### Ethics statement, sample collection, and RNA preparation

Samples were collected from 131 previously untreated CLL patients at The University of Texas M.D. Anderson Cancer Center (Houston, TX, USA) after obtaining written informed consent. The study was approved by The University of Texas M.D. Anderson Cancer Center Institutional Review Board and conducted according to the principles expressed in the Declaration of Helsinki. Clinical and routine laboratory data were obtained from review of the medical records. Peripheral blood was collected and processed as described previously [47]. Total RNA was extracted using two rounds of guanidine isothiocyanate/ phenol:chloroform extraction (TRIzol® Reagent, Life Technologies, Inc., Gaithersburg, MD) according to the manufacturer's instructions. Total RNA was further purified using RNeasy spin columns (Qiagen, Valencia, CA) and its quality assessed by agarose gel electrophoresis. Total RNA was reverse transcribed using random hexamers and a First-Strand cDNA Synthesis kit (Amersham Pharmacia Biotech, Inc., Piscataway, NJ). The cDNA was used for all subsequent PCR assays.

### Evaluation of the *IGHV* somatic mutation status

The somatic mutation status of the *IGHV* genes was assessed as described previously, with minor modifications [47]. Briefly, we amplified cDNA using a mixture of six 5′ *V_H_* primers that amplify all seven *V_H_* families and a 3′ constant region primer (*Cμ*) in the presence of reaction buffer, deoxynucleotide triphosphates (2.5 µM), and HotStar Taq DNA polymerase (Qiagen Sciences). Following incubation at 94°C for 15 minutes cDNA was amplified for 30 cycles (94°C for 1 minute, 56°C for 1 minute, and 72°C for 1 minute). In cases that failed to amplify using this strategy, we used a mixture of *V_H_* Framework 1 primers and a 3′ *J_H_* consensus primer (5′-AACTGAGGAGACGGTGACC-3′) Two independent PCR reactions were performed for each sample. The gel-purified PCR products were sequenced directly using the 3′ PCR primer and an ABI 3700 or 3730 DNA Analyzer (Applied Biosystems, Branchburg, NJ). In order to determine the degree of *IGHV* somatic mutation, patient sequences were aligned with germline sequences in VBASE II [48]. The somatic mutation status was designated as unmutated if there were <2% mutations (>98% homology to germline sequences), or as mutated if there were ≥2% mutations (≤98% homology to germline sequences) compared with germline sequences [49].

### Assessment of ZAP70 protein expression

Expression of ZAP70 was assessed either by immunohistochemistry or flow cytometry. Immunohistochemical staining was performed using routinely fixed and processed paraffin-embedded tissue sections of bone marrow core biopsy and/or aspirate clot specimens and a specific monoclonal antibody (Upstate Cell Signaling Systems, Lake Placid, NY, USA) [8], [11]. The flow cytometry assay for ZAP70 was performed by the Chronic Lymphocytic Leukemia Research Consortium laboratory as described previously [50].

### Design and production of the custom QRT-PCR microfluidics card

We printed 384-well custom microfluidics cards (MF) with gene-specific forward and reverse primers and TaqMan® probes to assess candidate biomarkers, and performed semi-quantitative real-time polymerase chain reaction (QRT-PCR) assays, as described previously [13], [14]. Also included were five endogenous control genes, *18S rRNA*, *GAPD*, *PGK1*, *GUSB*, and *ECE-1*, that spanned the dynamic range of the microfluidics cards. We used three different cards, designated A, B, and C, printed with candidate biomarkers of prognosis in CLL. Card A was printed with 88 candidate biomarkers of *IGHV* somatic mutation status [1], [2], [3], [4], [12], [13]. Card B was printed with 91 candidate biomarkers associated with prognostic factors other than *IGHV* somatic mutation status (**Table S3**) [5], [6], [7], [14], [15], [16], [17], [18]. Card C, which was used for validation, was printed with 37 candidate biomarkers that appeared to predict TTT based on a preliminary analysis of cards A and B, and the five endogenous control genes (**Table S4**). We assayed 65 training patient samples on cards A and B, and 66 validation patient samples on card C.

### Statistical Methods

The MF-QRT-PCR assay data were processed as described previously [13], [14]. Briefly, data from each card were quantified separately using the SDS 2.1 software package (Applied Biosystems, Carlsbad, CA) with an automatic baseline and a manual threshold of 0.10 to record the cycle thresholds, C_T_. Next, ΔC_T_ values were computed by subtracting the mean of the five endogenous control genes: *18S rRNA*, *GAPD*, *PGK1*, *GUSB*, and *ECE-1*. Statistical analyses were performed in version 2.11 of R, a freely available statistical software package (http://cran.r-project.org/). Differences in clinical and laboratory covariates between training and validation datasets were tested using unequal-variance two-sample t-tests for continuous variables and Fisher's Exact Test for dichotomous (binary) variables.

Kaplan-Meier estimates were used to plot survival curves. Univariate time-to-event analyses (for TTT and for OS) were conducted using Cox proportional hazards models and tested for significance using the log rank test. In order to select the best sets of predictors in multivariate models, we began with a Cox proportional hazards model that incorporated all variables. We then used a stepwise backward-forward algorithm to optimize either the Akaike Information Criterion (AIC) or the Bayes Information Criterion (BIC) [19]. The best multivariate models were selected based on AIC. (Models selected using BIC were almost always identical to those selected by AIC; when they differed, the AIC model included an additional factor, which we chose to retain.) We extensively cross-validated potential models on the training set before applying the best potential model to the validation dataset. The complete computer scripts used to analyze the data are available at http://bioinformatics.mdanderson.org/Supplements/Microfluidics/Prognosis [21].

## Supporting Information

Figure S1 **Flow diagram of the complete statistical analysis.**
(JPG)

Table S1 (A) Ability of individual genes to predict overall survival in the training dataset. (B) Genes ability to predict time-to-treatment, after accounting for clinical variables. (C) Genes ability to predict overall survival, after accounting for clinical variables.
(DOC)

Table S2 **Gene prognostic (GP) scores from 12 predictive models on the training set samples.**
(DOC)

Table S3 **Genes printed on microfluidics Card B.**
(DOC)

Table S4 **Genes printed on microfluidics Card C.**
(DOC)
