# Supplementary figures and images for: A Two-Gene Signature, SKI and SLAMF1, Predicts Time-to-Treatment in Previously Untreated Patients with Chronic Lymphocytic Leukemia
Source: PLoS One. 2011 Dec 14;6(12):e28277. doi: 10.1371/journal.pone.0028277 (PMC3237436; doi:10.1371/journal.pone.0028277)

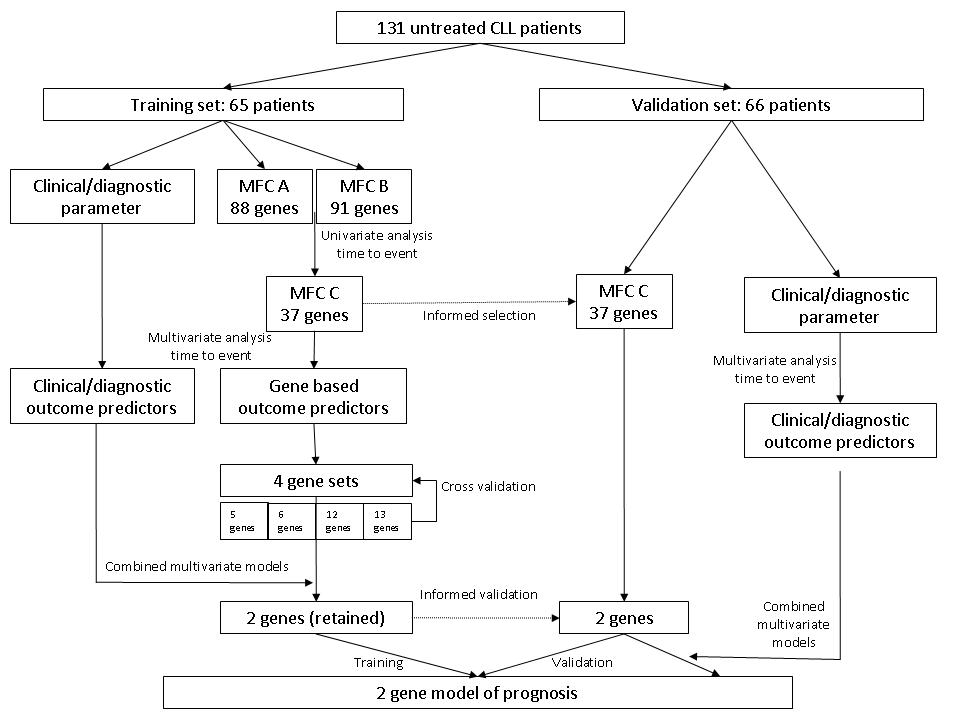

Supplement: Figure S1 — Flow diagram of the complete statistical analysis. (JPG) [file pone.0028277.s001.jpg]
