# Supplementary material for: A Two-Gene Signature, SKI and SLAMF1, Predicts Time-to-Treatment in Previously Untreated Patients with Chronic Lymphocytic Leukemia
Source: PLoS One. 2011 Dec 14;6(12):e28277. doi: 10.1371/journal.pone.0028277 (PMC3237436; doi:10.1371/journal.pone.0028277)
Supplement: Table S1 — (A) Ability of individual genes to predict overall survival in the training dataset. (B) Genes ability to predict time-to-treatment, after accounting for clinical variables. (C) Genes ability to predict overall survival, after accounting for clinical variables. (DOC) [file pone.0028277.s002.doc]

**Table S1 (A)**

**Ability of individual genes to predict overall survival in the training dataset.**

|  | **Cox proportional hazards**  **Log-rank p-values** | | | | **Cross-validation**  **% of times selected** | | |
| --- | --- | --- | --- | --- | --- | --- | --- |
|  | **Dichot.** | **Cont.** | **Dichot.** | **Cont.** | **Univ.** | **Multiv.** | **Multiv.** |
|  | **Diagn.** | **Diagn.** | **Diagn.** | **Diagn.** |  | **Diagn.** | **Diagn.** |
| CD14 | 0.04503 | 0.01569 | 0.06812 | 0.03486 | 27.0 | 10.3 | 7.0 |
| WSB2 | 0.10437 | 0.04194 | 0.04944 | 0.00983 | 15.3 | 2.0 | 3.3 |
| TNFRSF8 | 0.03513 | 0.10668 | 0.03267 | 0.06457 | 14.7 | 9.0 | 10.3 |
| NT5C2 | 0.05674 | 0.06056 | 0.02592 | 0.02885 | 13.3 | 6.0 | 9.3 |
| ZBTB20 | 0.07032 | 0.13435 | 0.02458 | 0.05040 | 7.3 | 1.3 | 1.7 |
| ANXA2 | 0.05389 | 0.09354 | 0.03798 | 0.09968 | 7.3 | 4.7 | 3.0 |
| BANK1 | 0.22501 | 0.14066 | 0.08838 | 0.05491 | 6.3 | 1.7 | 3.3 |
| CCL5 | 0.22956 | 0.08942 | 0.12984 | 0.03219 | 5.7 | 5.3 | 5.7 |
| EGR3 | 0.07073 | 0.08990 | 0.07444 | 0.06128 | 5.3 | 0.0 | 0.7 |
| SLAMF1 | 0.07113 | 0.13460 | 0.08488 | 0.14518 | 5.0 | 1.7 | 1.3 |
| GFI1 | 0.17011 | 0.11378 | 0.08130 | 0.06513 | 4.0 | 0.0 | 0.7 |
| SKI | 0.11984 | 0.12070 | 0.19367 | 0.12081 | 3.7 | 0.7 | 0.7 |
| ZAP70 | 0.15661 | 0.19635 | 0.09309 | 0.09034 | 3.0 | 0.3 | 1.0 |
| SEPT10 | 0.31242 | 0.18747 | 0.10538 | 0.04668 | 2.3 | 0.0 | 0.7 |
| NUDC | 0.08022 | 0.16679 | 0.14331 | 0.35741 | 2.0 | 1.0 | 0.3 |
| FGL2 | 0.40400 | 0.06015 | 0.24784 | 0.04062 | 2.0 | 1.0 | 1.7 |
| MLXIP | 0.09176 | 0.21070 | 0.13895 | 0.26818 | 1.3 | 1.0 | 0.7 |
| LDOC1 | 0.19249 | 0.35300 | 0.07751 | 0.13598 | 0.7 | 0.0 | 0.0 |
| FLNB | 0.46486 | 0.15787 | 0.25617 | 0.08001 | 0.7 | 0.0 | 0.0 |
| RIOK2 | 0.47638 | 0.38815 | 0.52041 | 0.50573 | 0.3 | 0.3 | 0.3 |
| COBLL1 | 0.33083 | 0.17936 | 0.17765 | 0.04214 | 0.3 | 0.0 | 0.0 |
| ATRX | 0.38658 | 0.86573 | 0.57260 | 0.60658 | 0.0 | 0.0 | 0.0 |
| GZMK | 0.89044 | 0.82761 | 0.78645 | 0.65688 | 0.0 | 0.0 | 0.0 |
| BCL7A | 0.78689 | 0.73583 | 0.36072 | 0.37923 | 0.0 | 0.0 | 0.0 |
| CRY1 | 0.68209 | 0.72146 | 0.53780 | 0.51581 | 0.0 | 0.0 | 0.0 |
| AICDA | 0.20748 | 0.67217 | 0.16443 | 0.51002 | 0.0 | 0.0 | 0.0 |
| TRIB2 | 0.61535 | 0.66571 | 0.44992 | 0.61334 | 0.0 | 0.0 | 0.0 |
| LASS6 | 0.77360 | 0.61048 | 0.69405 | 0.52760 | 0.0 | 0.0 | 0.0 |
| ATF4 | 0.37821 | 0.59133 | 0.48741 | 0.23770 | 0.0 | 0.0 | 0.0 |
| BLNK | 0.52158 | 0.57897 | 0.48516 | 0.70928 | 0.0 | 0.0 | 0.0 |
| CD86 | 0.60589 | 0.53040 | 0.31212 | 0.29391 | 0.0 | 0.0 | 0.0 |
| FGFR1 | 0.81505 | 0.51831 | 0.83677 | 0.81201 | 0.0 | 0.0 | 0.0 |
| TPST2 | 0.14111 | 0.37393 | 0.06515 | 0.15809 | 0.0 | 0.0 | 0.0 |
| P2RX1 | 0.99735 | 0.18513 | 0.99726 | 0.11072 | 0.0 | 0.0 | 0.0 |
| NRIP1 | 0.65204 | 0.16731 | 0.45520 | 0.18371 | 0.0 | 0.0 | 0.0 |
| OAS3 | 0.99949 | 0.16718 | 0.89695 | 0.18884 | 0.0 | 0.0 | 0.0 |
| LPL | 0.27333 | 0.15751 | 0.18570 | 0.06956 | 0.0 | 0.0 | 0.0 |

**Table S1 (B)**

**Genes ability to predict time-to-treatment, after accounting for clinical variables.**

|  | **Cox proportional hazards**  **Log-rank p-values** | | | | **Cross-validation**  **% of times selected** | | |
| --- | --- | --- | --- | --- | --- | --- | --- |
|  | **Dichot.** | **Cont.** | **Dichot.** | **Cont.** | **Univ.** | **Multiv.** | **Multiv.** |
|  | **Diagn.** | **Diagn.** | **Sample** | **Sample** |  | **Diagn.** | **Sample** |
| SLAMF1 | 0.07346 | 0.07934 | 0.16393 | 0.02484 | 8.7 | 7.7 | 7.7 |
| NRIP1 | 0.34321 | 0.61458 | 0.01818 | 0.02499 | 1.3 | 0.7 | 1.0 |
| SKI | 0.07843 | 0.08452 | 0.26328 | 0.10153 | 2.7 | 2.7 | 2.7 |
| NT5C2 | 0.07868 | 0.16157 | 0.11650 | 0.17149 | 4.3 | 4.3 | 4.3 |
| CD14 | 0.26590 | 0.21904 | 0.12944 | 0.07724 | 1.3 | 1.0 | 1.3 |
| FGFR1 | 0.33527 | 0.42532 | 0.03905 | 0.13735 | 0.7 | 0.3 | 0.0 |
| BANK1 | 0.81873 | 0.37826 | 0.35064 | 0.03780 | 0.0 | 0.0 | 0.0 |
| CD86 | 0.42221 | 0.51092 | 0.22431 | 0.09829 | 0.7 | 0.3 | 0.3 |
| FLNB | 0.09950 | 0.43235 | 0.20449 | 0.58346 | 0.3 | 0.3 | 0.3 |
| CRY1 | 0.07698 | 0.30233 | 0.84680 | 0.34699 | 0.0 | 0.0 | 0.0 |
| OAS3 | 0.69578 | 0.40512 | 0.60170 | 0.05775 | 0.3 | 0.3 | 0.3 |
| ATRX | 0.73242 | 0.32253 | 0.18806 | 0.25086 | 0.0 | 0.0 | 0.0 |
| P2RX1 | 0.51347 | 0.45467 | 0.15344 | 0.44770 | 0.0 | 0.0 | 0.0 |
| GZMK | 0.42879 | 0.30817 | 0.78261 | 0.16385 | 0.0 | 0.0 | 0.0 |
| LPL | 0.10190 | 0.32927 | 0.75060 | 0.67382 | 0.0 | 0.0 | 0.0 |
| ATF4 | 0.19149 | 0.40289 | 0.77916 | 0.43167 | 0.0 | 0.0 | 0.0 |
| EGR3 | 0.88550 | 0.15514 | 0.89834 | 0.28570 | 0.0 | 0.0 | 0.0 |
| MLXIP | 0.64224 | 0.67302 | 0.19673 | 0.42424 | 0.0 | 0.0 | 0.0 |
| ZAP70 | 0.15409 | 0.90545 | 0.31380 | 0.88321 | 0.0 | 0.0 | 0.0 |
| ZBTB20 | 0.15901 | 0.38752 | 0.90312 | 0.89761 | 0.0 | 0.0 | 0.0 |
| SEPT10 | 0.50227 | 0.42199 | 0.99384 | 0.32947 | 0.0 | 0.0 | 0.0 |
| ANXA2 | 0.71974 | 0.41127 | 0.53409 | 0.44647 | 0.0 | 0.0 | 0.0 |
| CCL5 | 0.35318 | 0.40347 | 0.86959 | 0.81829 | 0.0 | 0.0 | 0.0 |
| RIOK2 | 0.20833 | 0.73012 | 0.77102 | 0.89548 | 0.0 | 0.0 | 0.0 |
| TRIB2 | 0.90857 | 0.92309 | 0.57781 | 0.25030 | 0.0 | 0.0 | 0.0 |
| BCL7A | 0.45060 | 0.99011 | 0.81132 | 0.33731 | 0.0 | 0.0 | 0.0 |
| GFI1 | 0.37195 | 0.72170 | 0.76478 | 0.62095 | 0.0 | 0.0 | 0.0 |
| WSB2 | 0.42075 | 0.86100 | 0.44686 | 0.92329 | 0.0 | 0.0 | 0.0 |
| LASS6 | 0.92345 | 0.84269 | 0.74473 | 0.27888 | 0.0 | 0.0 | 0.0 |
| BLNK | 0.58268 | 0.35571 | 0.94226 | 0.83057 | 0.0 | 0.0 | 0.0 |
| AICDA | 0.45275 | 0.58518 | 0.71118 | 0.98214 | 0.0 | 0.0 | 0.0 |
| TPST2 | 0.87459 | 0.64704 | 0.84238 | 0.39754 | 0.0 | 0.0 | 0.0 |
| NUDC | 0.33833 | 0.69967 | 0.90763 | 0.99802 | 0.0 | 0.0 | 0.0 |
| COBLL1 | 0.79349 | 0.97165 | 0.33213 | 0.92943 | 0.0 | 0.0 | 0.0 |
| FGL2 | 0.83173 | 0.89566 | 0.59236 | 0.70289 | 0.0 | 0.0 | 0.0 |
| TNFRSF8 | 0.92081 | 0.77652 | 0.96517 | 0.48119 | 0.0 | 0.0 | 0.0 |
| LDOC1 | 0.99722 | 0.91849 | 0.90507 | 0.82529 | 0.0 | 0.0 | 0.0 |

**Table S1 (C)**

**Genes ability to predict overall survival, after accounting for clinical variables.**

|  | **Cox proportional hazards**  **Log-rank p-values** | | | | **Cross-validation**  **% of times selected** | | |
| --- | --- | --- | --- | --- | --- | --- | --- |
|  | **Dichot.** | **Cont.** | **Dichot.** | **Cont.** | **Univ.** | **Multiv.** | **Multiv.** |
|  | **Diagn.** | **Diagn.** | **Sample** | **Sample** |  | **Diagn.** | **Sample** |
| CRY1 | 0.08409 | 0.07732 | 0.04590 | 0.02665 | 20.7 | 1.7 | 1.0 |
| NT5C2 | 0.10606 | 0.06831 | 0.14380 | 0.06892 | 7.0 | 3.0 | 4.0 |
| CD14 | 0.13051 | 0.08136 | 0.15179 | 0.12784 | 6.0 | 5.3 | 5.3 |
| MLXIP | 0.04951 | 0.17494 | 0.12291 | 0.28718 | 1.7 | 1.0 | 0.7 |
| SLAMF1 | 0.20126 | 0.06740 | 0.25181 | 0.13531 | 7.7 | 5.3 | 3.7 |
| LASS6 | 0.24051 | 0.43423 | 0.05210 | 0.14221 | 0.0 | 0.0 | 0.0 |
| FGFR1 | 0.24129 | 0.10626 | 0.38654 | 0.15722 | 2.0 | 0.7 | 0.7 |
| WSB2 | 0.27856 | 0.37525 | 0.11951 | 0.14751 | 0.3 | 0.0 | 0.0 |
| EGR3 | 0.12553 | 0.21863 | 0.30999 | 0.32147 | 0.3 | 0.0 | 0.0 |
| CCL5 | 0.59606 | 0.35907 | 0.33252 | 0.12116 | 0.3 | 0.3 | 0.3 |
| NRIP1 | 0.33289 | 0.36786 | 0.07870 | 0.91960 | 0.0 | 0.0 | 0.0 |
| ATF4 | 0.68593 | 0.31918 | 0.86541 | 0.06480 | 0.3 | 0.0 | 0.0 |
| GFI1 | 0.34713 | 0.42659 | 0.25137 | 0.34750 | 0.0 | 0.0 | 0.0 |
| OAS3 | 0.63580 | 0.09176 | 0.98735 | 0.22461 | 0.0 | 0.0 | 0.0 |
| BCL7A | 0.15449 | 0.30974 | 0.60937 | 0.48080 | 0.0 | 0.0 | 0.0 |
| ANXA2 | 0.24621 | 0.58345 | 0.16200 | 0.83787 | 0.0 | 0.0 | 0.0 |
| TNFRSF8 | 0.19142 | 0.72229 | 0.24400 | 0.58397 | 1.7 | 1.7 | 1.7 |
| AICDA | 0.48953 | 0.54724 | 0.84253 | 0.11519 | 0.0 | 0.0 | 0.0 |
| NUDC | 0.20611 | 0.35312 | 0.49035 | 0.87927 | 0.3 | 0.3 | 0.0 |
| BLNK | 0.29099 | 0.39539 | 0.38591 | 0.70964 | 0.0 | 0.0 | 0.0 |
| TPST2 | 0.33570 | 0.88489 | 0.31386 | 0.39292 | 0.0 | 0.0 | 0.0 |
| ZBTB20 | 0.31962 | 0.72095 | 0.32222 | 0.68552 | 0.0 | 0.0 | 0.0 |
| SKI | 0.35748 | 0.39441 | 0.77639 | 0.65758 | 0.0 | 0.0 | 0.0 |
| FGL2 | 0.96587 | 0.20621 | 0.56481 | 0.65725 | 0.0 | 0.0 | 0.0 |
| FLNB | 0.34186 | 0.71347 | 0.68980 | 0.46352 | 0.0 | 0.0 | 0.0 |
| COBLL1 | 0.70410 | 0.94353 | 0.46687 | 0.26883 | 0.0 | 0.0 | 0.0 |
| SEPT10 | 0.87035 | 0.68741 | 0.42200 | 0.36071 | 0.0 | 0.0 | 0.0 |
| BANK1 | 0.74490 | 0.65832 | 0.39251 | 0.55838 | 0.0 | 0.0 | 0.0 |
| TRIB2 | 0.73886 | 0.51903 | 0.75544 | 0.55898 | 0.0 | 0.0 | 0.0 |
| ATRX | 0.87585 | 0.55004 | 0.82240 | 0.49201 | 0.0 | 0.0 | 0.0 |
| GZMK | 0.48553 | 0.84225 | 0.52038 | 0.96540 | 0.0 | 0.0 | 0.0 |
| RIOK2 | 0.72308 | 0.59261 | 0.72384 | 0.68219 | 0.0 | 0.0 | 0.0 |
| P2RX1 | 0.99746 | 0.36408 | 0.99761 | 0.66471 | 0.0 | 0.0 | 0.0 |
| CD86 | 0.59720 | 0.99330 | 0.65339 | 0.77243 | 0.0 | 0.0 | 0.0 |
| ZAP70 | 0.57864 | 0.70990 | 0.98941 | 0.74000 | 0.3 | 0.3 | 0.3 |
| LDOC1 | 0.65037 | 0.88357 | 0.80787 | 0.87401 | 0.0 | 0.0 | 0.0 |
| LPL | 0.98450 | 0.99814 | 0.68680 | 0.87691 | 0.0 | 0.0 | 0.0 |
