# Supplementary material for: A Two-Gene Signature, SKI and SLAMF1, Predicts Time-to-Treatment in Previously Untreated Patients with Chronic Lymphocytic Leukemia
Source: PLoS One. 2011 Dec 14;6(12):e28277. doi: 10.1371/journal.pone.0028277 (PMC3237436; doi:10.1371/journal.pone.0028277)
Supplement: Table S2 — Gene prognostic (GP) scores from 12 predictive models on the training set samples. (DOC) [file pone.0028277.s003.doc]

**Table S2: Gene prognostic (GP) scores from 12 predictive models on the training set samples.**

|  | **M6**  **AIC** | **M6**  **BIC** | **M6,SAM AIC** | **M5**  **AIC** | **M6,SAM BIC** | **M5** | **M12 AIC** | **M6** | **M13 AIC** | **M12** | **M13** | **SKI+ SLAMF1** |
| --- | --- | --- | --- | --- | --- | --- | --- | --- | --- | --- | --- | --- |
| **CL001** | -0.7311 | -0.8578 | -0.9813 | -0.9449 | -0.8156 | -0.9860 | -1.0065 | -0.7740 | -0.5899 | -0.6960 | -0.3901 | -0.7629 |
| **CL002** | -0.1500 | -0.2688 | 0.1574 | 0.3271 | -0.1370 | 0.3790 | -0.3419 | -0.2369 | -0.6709 | -0.1364 | -0.3193 | -0.2905 |
| **CL007** | -0.9771 | -1.1362 | -1.0225 | -1.3218 | -1.0021 | -1.2719 | -1.2909 | -1.0068 | -1.3027 | -1.0711 | -1.0407 | -1.1373 |
| **CL008** | 0.5228 | 0.3335 | 0.2824 | 0.5184 | 0.1956 | 0.2138 | -0.0728 | 0.1626 | 0.5156 | 0.1718 | 0.3081 | 0.0206 |
| **CL010** | -0.3188 | -0.7242 | -0.1069 | -0.1411 | -0.5994 | -0.3002 | 0.2006 | -0.6934 | 0.5838 | 0.1044 | 0.2652 | -1.2546 |
| **CL012** | -0.4095 | -0.3702 | -0.5177 | -0.1747 | -0.3925 | -0.1806 | -0.0922 | -0.3484 | 0.2834 | 0.8410 | 0.5490 | -0.7662 |
| **CL013** | -1.0211 | -1.1538 | -1.4194 | -1.2347 | -1.1100 | -1.2807 | -1.8245 | -1.2090 | -2.1693 | -2.1001 | -2.1989 | -0.9386 |
| **CL014** | -1.1555 | -1.2815 | -1.6326 | -0.9771 | -1.2594 | -1.0587 | -1.4062 | -1.2410 | -1.3521 | -1.4583 | -1.1937 | -1.2185 |
| **CL016** | 0.2296 | 0.0426 | -0.3627 | -0.0223 | -0.1708 | -0.4090 | -0.5497 | -0.2009 | -0.5000 | -0.9620 | -0.9760 | -0.2966 |
| **CL022** | -0.4619 | -0.5361 | -0.3785 | -0.6290 | -0.4435 | -0.5688 | -0.5186 | -0.4132 | -0.9654 | -0.4948 | -1.1614 | -0.5754 |
| **CL026** | -0.8980 | -1.1089 | -1.1283 | -0.9491 | -1.0322 | -1.0183 | -1.0156 | -0.9801 | -1.1247 | -1.3668 | -1.1949 | -1.0844 |
| **CL027** | -1.0440 | -1.1049 | -1.3271 | -1.2538 | -1.0319 | -1.2108 | -0.8939 | -1.0737 | -1.6343 | -1.5069 | -1.6127 | -0.8701 |
| **CL029** | -0.4237 | -0.4704 | -0.3951 | -0.6196 | -0.3901 | -0.5534 | -0.8970 | -0.3512 | -0.8174 | -0.9222 | -0.8082 | -0.3472 |
| **CL033** | 0.3894 | 0.5037 | 0.6745 | 0.4512 | 0.5092 | 0.5450 | -0.0870 | 0.3794 | -0.0558 | 0.0368 | -0.0286 | 0.3120 |
| **CL037** | -1.1737 | -1.2354 | -1.2709 | -1.1958 | -1.0855 | -1.0681 | -1.8139 | -1.0603 | -2.2822 | -1.5742 | -1.9459 | -0.9733 |
| **CL038** | 0.4653 | 0.4308 | 1.0441 | 0.4531 | 0.5333 | 0.5561 | -0.4097 | 0.4691 | -0.1958 | 0.5312 | 0.0023 | 0.1402 |
| **CL039** | -0.8099 | -0.5609 | -1.0117 | -1.3747 | -0.5412 | -1.1485 | -0.2544 | -0.5168 | -0.1609 | -0.3333 | -0.1802 | -0.3298 |
| **CL040** | -0.3124 | -0.3899 | -0.6803 | -0.1140 | -0.4434 | -0.2470 | -0.4864 | -0.3686 | -0.5483 | -0.6090 | -0.4093 | -0.2753 |
| **CL041** | -0.2938 | -0.0912 | -0.3589 | -0.6836 | -0.0878 | -0.5155 | -0.2484 | -0.1070 | -0.1403 | -0.6697 | -0.1139 | 0.1252 |
| **CL042** | 0.6360 | 0.4569 | 0.7566 | 0.5906 | 0.4599 | 0.4633 | 0.5186 | 0.4275 | 0.4465 | 0.4523 | 0.5756 | 0.6591 |
| **CL043** | 0.3485 | 0.2657 | 0.3731 | -0.0113 | 0.2247 | -0.1069 | -0.3780 | 0.2895 | 0.1058 | -0.3452 | -0.2177 | 0.0581 |
| **CL044** | 0.6133 | 0.6746 | 0.7581 | 0.6625 | 0.6194 | 0.6444 | 0.7153 | 0.6214 | 1.4352 | 0.3932 | 1.3533 | 0.5189 |
| **CL046** | 0.0020 | 0.0518 | 0.4053 | 0.0483 | 0.1551 | 0.2104 | -0.0397 | 0.1673 | 0.1967 | -0.2895 | 0.0163 | -0.1174 |
| **CL048** | -0.0064 | -0.0123 | -0.6160 | -0.1289 | -0.2181 | -0.3769 | -0.5369 | -0.2140 | -0.4685 | -0.7927 | -0.6410 | -0.2128 |
| **CL049** | -0.8196 | -0.7584 | -1.3919 | -0.9839 | -0.8183 | -1.0107 | -0.9692 | -0.8388 | -1.6823 | -1.6274 | -1.8243 | -0.4661 |
| **CL050** | 0.6539 | 0.5898 | 1.2132 | 0.4497 | 0.6739 | 0.5157 | 0.5801 | 0.7509 | 0.5202 | 0.5375 | 0.2573 | 0.3773 |
| **CL051** | 0.1182 | 0.4200 | 0.1558 | 0.2123 | 0.3940 | 0.4035 | 1.2502 | 0.3576 | 0.6645 | 1.0089 | 0.6484 | 0.7086 |
| **CL052** | -0.0446 | -0.1187 | -0.2587 | 0.0876 | -0.1590 | -0.0229 | 0.7610 | -0.1170 | 0.4952 | 0.5126 | 0.7247 | -0.0110 |
| **CL053** | 0.4385 | 0.6339 | 0.8838 | 0.6503 | 0.6917 | 0.8618 | 1.7280 | 0.7198 | 1.7025 | 1.5961 | 1.6709 | 0.6902 |
| **CL054** | -0.0102 | 0.4299 | -0.1328 | 0.0218 | 0.3147 | 0.2131 | -0.0729 | 0.3145 | -0.0841 | -0.3091 | -0.4272 | 0.3264 |
| **CL056** | 0.4059 | 0.6897 | 0.8432 | 0.2013 | 0.7289 | 0.4725 | 0.1424 | 0.7695 | 0.3527 | 0.1447 | 0.1994 | 0.6733 |
| **CL057** | -0.4231 | -0.6255 | -0.5060 | -0.5012 | -0.5633 | -0.5781 | -0.5150 | -0.5343 | -0.5179 | -0.3885 | -0.4010 | -0.5935 |
| **CL058** | 0.0592 | -0.0431 | 0.2825 | -0.0827 | 0.0381 | -0.0559 | 0.5437 | 0.1474 | 0.5824 | 0.2330 | 0.3882 | -0.0097 |
| **CL059** | 0.1578 | -0.0199 | -0.0537 | -0.1387 | -0.0864 | -0.3407 | 0.0230 | -0.0711 | 0.3084 | -0.6020 | -0.1343 | -0.1203 |
| **CL061** | 0.6696 | 0.4041 | 0.6317 | 0.8547 | 0.3192 | 0.5509 | 0.1701 | 0.3035 | 0.7854 | 0.9536 | 0.9496 | 0.0480 |
| **CL062** | -0.4621 | -0.5450 | -0.4252 | -0.3001 | -0.4217 | -0.2247 | -0.1839 | -0.4851 | 0.3478 | 0.0196 | 0.5233 | -0.0735 |
| **CL063** | -0.1537 | -0.4355 | -0.9628 | -0.1721 | -0.6286 | -0.6181 | -0.8540 | -0.6198 | -1.2384 | -0.7631 | -1.1485 | -0.4852 |
| **CL066** | -0.5076 | -0.0208 | 0.1265 | -0.1573 | 0.1754 | 0.4306 | -0.2254 | 0.0837 | -0.6774 | 0.2422 | -0.5372 | 0.1072 |
| **CL067** | -0.1040 | -0.1114 | -1.1687 | -0.1186 | -0.3971 | -0.4727 | -0.8993 | -0.3978 | -1.9455 | -1.6353 | -2.5031 | 0.2675 |
| **CL068** | 0.2997 | 0.3355 | 0.8665 | 0.3597 | 0.4709 | 0.5500 | 0.1381 | 0.4599 | 0.0233 | 0.0015 | -0.0486 | 0.3570 |
| **CL069** | -0.1798 | 0.3195 | 0.0590 | -0.3634 | 0.3896 | 0.0997 | 0.6965 | 0.4353 | 0.1211 | 0.8899 | 0.4582 | 0.9213 |
| **CL070** | 1.0405 | 1.0320 | 2.0079 | 0.7728 | 1.1853 | 0.9692 | 0.7303 | 1.1641 | 1.0172 | 0.8308 | 1.0099 | 0.8119 |
| **CL071** | 0.0517 | -0.2382 | 0.4734 | -0.0252 | -0.0862 | -0.0549 | -0.9023 | -0.0128 | -0.7911 | -0.1782 | -0.6048 | -0.4581 |
| **CL072** | -0.0803 | 0.0385 | 0.1072 | 0.1435 | 0.1070 | 0.3059 | 0.7239 | 0.0576 | 0.3089 | 0.5372 | 0.4934 | 0.2549 |
| **CL073** | 0.5220 | 0.8239 | 0.6220 | 0.5300 | 0.7666 | 0.6887 | 1.3380 | 0.7380 | 0.7492 | 1.2605 | 0.7031 | 1.1150 |
| **CL075** | 0.7888 | 0.6072 | 0.6089 | 0.5667 | 0.4638 | 0.2700 | 1.1187 | 0.5694 | 1.6720 | 1.7112 | 1.7460 | 0.4170 |
| **CL076** | 0.3141 | 0.8085 | 0.2363 | 0.4039 | 0.6655 | 0.6016 | 0.6559 | 0.6518 | 0.9575 | 0.7932 | 1.0738 | 0.7956 |
| **CL078** | -0.6798 | -0.7839 | -0.9452 | -0.0986 | -0.7568 | -0.1700 | 0.3579 | -0.7934 | 0.0975 | 0.2895 | 0.4024 | -0.6936 |
| **CL080** | 0.5433 | 0.7534 | 0.7474 | 0.8130 | 0.7168 | 0.9199 | 1.0626 | 0.6521 | 1.0474 | 0.8160 | 0.9244 | 0.7688 |
| **CL081** | 0.0728 | -0.1645 | 0.5554 | 0.0542 | 0.0062 | 0.0839 | -0.7088 | -0.0276 | -0.7610 | -0.0972 | -0.5163 | -0.2600 |
| **CL088** | 0.5094 | 0.5750 | 0.6494 | 0.3819 | 0.5458 | 0.4034 | 0.5072 | 0.5604 | 0.6475 | 0.4825 | 0.4565 | 0.5945 |
| **CL090** | 0.6358 | 0.6668 | 0.2637 | 0.7052 | 0.4610 | 0.4806 | 0.4883 | 0.4957 | 0.6089 | -0.2775 | 0.1004 | 0.6146 |
| **CL092** | 0.1157 | 0.3981 | 0.3693 | 0.5509 | 0.4237 | 0.7789 | 0.2055 | 0.4271 | 0.4595 | 0.3931 | 0.5958 | 0.4626 |
| **CL093** | 0.3995 | 0.2302 | 0.2739 | 0.5555 | 0.1648 | 0.3453 | 0.4699 | 0.2182 | 0.6056 | 0.6719 | 0.9224 | 0.2015 |
| **CL095** | -0.2024 | -0.1227 | -0.4040 | -0.2982 | -0.1644 | -0.2868 | -0.2542 | -0.1613 | -0.1166 | -0.4355 | -0.2609 | -0.0256 |
| **CL097** | 0.0431 | 0.3530 | 0.3522 | -0.2414 | 0.4067 | 0.0670 | 0.4938 | 0.3943 | 0.4455 | 0.4776 | 0.7744 | 0.4868 |
| **CL099** | 0.3232 | 0.0704 | -0.0138 | 0.8315 | -0.0436 | 0.4866 | 0.5907 | 0.0098 | 0.5394 | 0.7490 | 0.6061 | -0.0212 |
| **CL100** | 0.3179 | 0.2486 | -0.7114 | 0.7253 | -0.1185 | 0.2163 | 0.3319 | -0.1281 | 0.4830 | 0.4904 | 0.4627 | 0.0557 |
| **CL102** | 0.1612 | 0.1248 | -0.0743 | -0.0397 | 0.0432 | -0.1582 | -0.4349 | 0.0131 | -0.5454 | -0.9569 | -0.8964 | 0.2015 |
| **CL103** | 0.2770 | 0.4357 | 0.4176 | 0.6285 | 0.4286 | 0.7274 | 1.1316 | 0.5090 | 1.3239 | 1.5032 | 1.6590 | 0.5400 |
| **CL104** | 0.2999 | 0.1364 | 0.7715 | 0.8756 | 0.2618 | 0.8855 | 1.3670 | 0.2936 | 1.2617 | 1.6176 | 1.3503 | 0.0220 |
| **CL107** | 0.4272 | 0.2500 | 0.6855 | -0.0464 | 0.2803 | -0.1216 | -0.6215 | 0.2599 | -0.1267 | -0.4716 | -0.2514 | -0.0344 |
| **CL109** | 0.0366 | -0.1253 | -0.0253 | -0.4346 | -0.1245 | -0.5379 | -0.3475 | -0.2121 | -0.4904 | -1.0464 | -0.9361 | -0.1340 |
| **CL110** | 0.6739 | 0.7412 | 1.2814 | 1.0027 | 0.8214 | 1.1432 | 0.5377 | 0.7114 | 0.7614 | 0.9954 | 1.0111 | 0.5789 |
| **CL119** | 0.2907 | 0.5396 | 0.3468 | 0.3486 | 0.4868 | 0.4712 | 1.5744 | 0.6095 | 1.4988 | 1.8274 | 1.7410 | 0.6047 |
