# Supplementary material for: A Two-Gene Signature, SKI and SLAMF1, Predicts Time-to-Treatment in Previously Untreated Patients with Chronic Lymphocytic Leukemia
Source: PLoS One. 2011 Dec 14;6(12):e28277. doi: 10.1371/journal.pone.0028277 (PMC3237436; doi:10.1371/journal.pone.0028277)
Supplement: Table S3 — Genes printed on microfluidics Card B. (DOC) [file pone.0028277.s004.doc]

**Table S3: Genes printed on microfluidics Card B.**

| **Symbol** | **Alternate Symbol** | **Unigene (build 171)** | **Chromosome** | **Gene Name** | **Entrez Gene** | **Microarray Study** |
| --- | --- | --- | --- | --- | --- | --- |
| HMGCS2 |  | Hs.59889 | 1p13-p12 | 3-hydroxy-3-methylglutaryl-Coenzyme A synthase 2 (mitochondrial) | 3158 | JELINEK |
| NASP |  | Hs.446206 | 1p34.1 | nuclear autoantigenic sperm protein (histone-binding) | 4678 | JELINEK |
| NUDC |  | Hs.263812 | 1p35-p34 | nuclear distribution gene C homolog (A. nidulans) | 10726 | JELINEK |
| MCL1 | TM, EAT | Hs.86386 | 1q21 | myeloid cell leukemia sequence 1 (BCL2-related) | 4170 | AALTO |
| MNDA |  | Hs.153837 | 1q22 | myeloid cell nuclear differentiation antigen | 4332 | AALTO |
| SELL | LAM1, CD62L | Hs.82848 | 1q23-q25 | selectin L (lymphocyte adhesion molecule 1) | 6402 | STRATOWA |
| CR2 | CD21 | Hs.73792 | 1q32 | complement component (3d/Epstein Barr virus) receptor 2 | 1380 | DURIG |
| FMOD | SLRR2E | Hs.442844 | 1q32 | fibromodulin | 2331 | VALLAT |
| ADPRT | PARP | Hs.177766 | 1q41-q42 | ADP-ribosyltransferase (NAD+; poly (ADP-ribose) polymerase) | 142 | AALTO |
| MAT2A | SAMS2 | Hs.77502 | 2p11.2 | methionine adenosyltransferase II, alpha | 4144 | VALLAT |
| REL |  | Hs.44313 | 2p13-p12 | v-rel reticuloendotheliosis viral oncogene homolog (avian) | 5966 | VALLAT |
| HTLF | FOXN2 | Hs.103126 | 2p22-p16 | human T-cell leukemia virus enhancer factor | 3344 | AALTO |
| TRIB2 |  | Hs.155418 | 2p25.1 | tribbles homolog 2 (Drosophila) | 28951 | DURIG |
| IL1B |  | Hs.126256 | 2q14 | interleukin 1, beta | 3553 | STRATOWA |
| GPR17 |  | Hs.46453 | 2q21 | G protein-coupled receptor 17 | 2840 | AALTO |
| ITGA4 | CD49D | Hs.528404 | 2q31-q32 | integrin, alpha 4 (antigen CD49D, alpha 4 subunit of VLA-4 receptor) | 3676 | DURIG |
| CLK1 | STY | Hs.433732 | 2q33 | CDC-like kinase 1 | 1195 | DURIG |
| FLNB |  | Hs.81008 | 3p14.3 | filamin B, beta (actin binding protein 278) | 2317 | DURIG, WIESTNER |
| CD38 | CD38 | Hs.174944 | 4p15 | CD38 antigen (p45) | 952 | DURIG |
| UGDH |  | Hs.28309 | 4p15.1 | UDP-glucose dehydrogenase | 7358 | JELINEK |
| LRPAP1 | RAP, MRAP | Hs.75140 | 4p16.3 | low density lipoprotein receptor-related protein associated protein 1 | 4043 | JELINEK |
| UGT2B15 |  | Hs.150207 | 4q13 | UDP glycosyltransferase 2 family, polypeptide B15 | 7366 | ABRUZZO |
| IL8 | IL8 | Hs.624 | 4q13-q21 | interleukin 8 | 3576 | AALTO, STRATOWA |
| GZMK | TRYP2 | Hs.277937 | 5q11-q12 | granzyme K (serine protease, granzyme 3; tryptase II) | 3003 | AALTO |
| MEF2C |  | Hs.368950 | 5q14 | MADS box transcription enhancer factor 2, polypeptide C (myocyte enhancer factor 2C) | 4208 | DURIG |
| CD14 |  | Hs.163867 | 5q22-q32 | CD14 antigen | 929 | AALTO |
| EGR1 | ZNF255 | Hs.326035 | 5q31.1 | early growth response 1 | 1958 | STRATOWA |
| TNIP1 | VAN, NAF-1 | Hs.355141 | 5q32-q33.1 | TNFAIP3 interacting protein 1 | 10318 | JELINEK |
| LCP2 | SLP76 | Hs.2488 | 5q33.1-qter | lymphocyte cytosolic protein 2 (SH2 domain containing leukocyte protein of 76kDa) | 3937 | AALTO |
| NPM1 | B23 | Hs.411098 | 5q35 | nucleophosmin (nucleolar phosphoprotein B23, numatrin) | 4869 | AALTO, ROSENWALD |
| HLA-DQA1 |  | Hs.387679 | 6p21.3 | major histocompatibility complex, class II, DQ alpha 1 | 3117 | VALLAT |
| MICB | PERB11.2 | Hs.211580 | 6p21.3 | MHC class I polypeptide-related sequence B | 4277 | JELINEK |
| DEK |  | Hs.110713 | 6p23 | DEK oncogene (DNA binding) | 7913 | AALTO |
| HIVEP1 | ZNF40 | Hs.405564 | 6p24-p22.3 | human immunodeficiency virus type I enhancer binding protein 1 | 3096 | AALTO |
| HIVEP2 |  | Hs.75063 | 6q23-q24 | human immunodeficiency virus type I enhancer binding protein 2 | 3097 | AALTO |
| T | TFT | Hs.389457 | 6q27 | T, brachyury homolog (mouse) | 6862 | JELINEK |
| TAF6 |  | Hs.289950 | 7q22.1 | TAF6 RNA polymerase II, TATA box binding protein (TBP)-associated factor, 80kDa | 6878 | JELINEK |
| SLC39A14 | KIAA0062 | Hs.301743 | 8p21.2 | solute carrier family 39 (zinc transporter), member 14 | 23516 | JELINEK |
| EGR3 | PILOT | Hs.74088 | 8p23-p21 | early growth response 3 | 1960 | DURIG |
| DKFZp564M1416 |  |  | 8q11.22-q11.23 | DKFZP564M1416 protein | 25869 | KLEIN |
| MYC |  | Hs.202453 | 8q24.12-q24.13 | v-myc myelocytomatosis viral oncogene homolog (avian) | 4609 | VALLAT |
| PBX3 |  | Hs.294101 | 9q33-q34 | pre-B-cell leukemia transcription factor 3 | 5090 | AALTO |
| FCN1 | FCNM | Hs.440898 | 9q34 | ficolin (collagen/fibrinogen domain containing) 1 | 2219 | JELINEK |
| STAM |  | Hs.441498 | 10p14-p13 | signal transducing adaptor molecule (SH3 domain and ITAM motif) 1 | 8027 | AALTO |
| IL2RA | CD25 | Hs.130058 | 10p15-p14 | interleukin 2 receptor, alpha | 3559 | AALTO |
| **Symbol** | **Alternate Symbol** | **Unigene (build 171)** | **Chromosome** | **Gene Name** | **Entrez Gene** | **Microarray Study** |
| ANXA11 |  | Hs.75510 | 10q23 | annexin A11 | 311 | JELINEK |
| ZNF289 | IRZ | Hs.436204 | 11p11.2-p11.12 | zinc finger protein 289, ID1 regulated | 84364 | JELINEK |
| CALCA | CGRP | Hs.37058 | 11p15.2-p15.1 | calcitonin/calcitonin-related polypeptide, alpha | 796 | AALTO |
| IFITM1 | CD225, LEU13 | Hs.458414 | 11p15.5 | interferon induced transmembrane protein 1 (9-27) | 8519 | JELINEK |
| BIRC2 | HIAP2 | Hs.289107 | 11q22 | baculoviral IAP repeat-containing 2 | 329 | VALLAT |
| BLR1 | CXCR5 | Hs.113916 | 11q23.3 | Burkitt lymphoma receptor 1, GTP binding protein (chemokine (C-X-C motif) receptor 5) | 643 | AALTO |
| KIAA1030 | KIAA1030 | Hs.204121 | 11q25 | KIAA1030 protein | 22997 | JELINEK |
| CD63 |  | Hs.445570 | 12q12-q13 | CD63 antigen (melanoma 1 antigen) | 967 | JELINEK |
| TEGT | BI-1 | Hs.35052 | 12q12-q13 | testis enhanced gene transcript (BAX inhibitor 1) | 7009 | JELINEK |
| ATP2B1 | PMCA1 | Hs.20952 | 12q21.3 | ATPase, Ca++ transporting, plasma membrane 1 | 490 | DURIG |
| NR4A1 | HMR, GFRP1 | Hs.1119 | 12q13 | nuclear receptor subfamily 4, group A, member 1 | 3164 | VALLAT |
| MONDOA | MIR | Hs.528339 | 12q21.31 | Mlx interactor | 22877 | JELINEK |
| TCF1 | HNF1, LFB1 | Hs.528674 | 12q22-qter | transcription factor 1, hepatic; LF-B1, hepatic nuclear factor (HNF1), albumin proximal factor | 6927 | AALTO |
| MTMR6 |  | Hs.79877 | 13q12 | myotubularin related protein 6 | 9107 | VALLAT |
| EVI2B |  | Hs.5509 | 17q11.2 | ecotropic viral integration site 2B | 2124 | AALTO |
| LCP1 | CP64, PLS2 | Hs.381099 | 13q14.3 | lymphocyte cytosolic protein 1 (L-plastin) | 3936 | AALTO |
| EBI2 |  | Hs.784 | 13q32.3 | Epstein-Barr virus induced gene 2 (lymphocyte-specific G protein-coupled receptor) | 1880 | AALTO |
| ISGF3G | p48 | Hs.1706 | 14q11.2 | interferon-stimulated transcription factor 3, gamma 48kDa | 10379 | DURIG |
| MED6 |  | Hs.167738 | 14q24.1 | mediator of RNA polymerase II transcription, subunit 6 homolog (yeast) | 10001 | JELINEK |
| EIF4A1 | DDX2A | Hs.129673 | 17p13 | eukaryotic translation initiation factor 4A, isoform 1 | 1973 | JELINEK |
| MAP2K3 | MEK3 | Hs.180533 | 17q11.2 | mitogen-activated protein kinase kinase 3 | 5606 | JELINEK |
| CCL5 | RANTES | Hs.489044 | 17q11.2-q12 | chemokine (C-C motif) ligand 5 | 6352 | JELINEK |
| NMT1 |  | Hs.346743 | 17q21.31 | N-myristoyltransferase 1 | 4836 | JELINEK |
| CD79B | IGB | Hs.89575 | 17q23 | CD79B antigen (immunoglobulin-associated beta) | 974 | AALTO |
| SLC16A3 | MCT3, MCT4 | Hs.386678 | 17q25 | solute carrier family 16 (monocarboxylic acid transporters), member 3 | 9123 | JELINEK |
| BCL2 |  | Hs.501181 Hs.79241 | 18q21.33 | B-cell CLL/lymphoma 2 | 596 | AALTO |
| GDF1 |  | Hs.412355 | 19p12 | growth differentiation factor 1 | 2657 | DURIG |
| MAG | GMA | Hs.1780 | 19q13.1 | myelin associated glycoprotein | 4099 | JELINEK |
| LISCH7 |  | Hs.312129 | 19q13.13 | liver-specific bHLH-Zip transcription factor | 51599 | JELINEK |
| PLA2G4C |  | Hs.18858 | 19q13.3 | phospholipase A2, group IVC (cytosolic, calcium-independent) | 8605 | JELINEK |
| NRIP1 | RIP140 | Hs.155017 | 21q11.2 | nuclear receptor interacting protein 1 | 8204 | DURIG |
| ITGB2 | CD18, MF17 | Hs.375957 | 21q22.3 | integrin, beta 2 (antigen CD18 (p95), lymphocyte function-associated antigen 1; macrophage antigen 1 (mac-1) beta subunit) | 3689 | STRATOWA |
| BIK |  | Hs.155419 | 22q13.31 | BCL2-interacting killer (apoptosis-inducing) | 638 | DURIG |
| NONO | P54, NMT55 | Hs.355861 | Xq13.1 | non-POU domain containing, octamer-binding | 4841 | JELINEK |
| ATRX | SHS, RAD54 | Hs.440734 | Xq13.1-q21.1 | alpha thalassemia/mental retardation syndrome X-linked (RAD54 homolog, S. cerevisiae) | 546 | JELINEK |
| IDS | SIDS, MPS2 | Hs.303154 | Xq28 | iduronate 2-sulfatase (Hunter syndrome) | 3423 | JELINEK |
