# Supplementary material for: A Two-Gene Signature, SKI and SLAMF1, Predicts Time-to-Treatment in Previously Untreated Patients with Chronic Lymphocytic Leukemia
Source: PLoS One. 2011 Dec 14;6(12):e28277. doi: 10.1371/journal.pone.0028277 (PMC3237436; doi:10.1371/journal.pone.0028277)
Supplement: Table S4 — Genes printed on microfluidics Card C. (DOC) [file pone.0028277.s005.doc]

**Table S4: Genes printed on microfluidics Card C.**

| *RN18S1* | BLNK | EGR3 | GZMK | NUDC | SLAMF1 |
| --- | --- | --- | --- | --- | --- |
| AICDA | CCL5 | FGFR1 | LASS6 | OAS3 | TNFRSF8 |
| ANXA2 | CD14 | FGL2 | LDOC1 | P2RX1 | TPST2 |
| ATF4 | CD86 | FLNB | LPL | *PGK1* | TRIB2 |
| ATRX | COBLL1 | *GAPDH* | MLXIP | RIOK2 | WSB2 |
| BANK1 | CRY1 | GFI1 | NRIP1 | SEPT10 | ZAP70 |
| BCL7A | *ECE1* | *GUSB* | NT5C2 | SKI | ZBTB20 |

Endogenous control (“housekeeping”) genes are marked in *italic* font.
